# Manumycin A Attenuates DSS-Induced Colitis in Mice via Anti-Inflammatory Effects Following Intraperitoneal Administration

**DOI:** 10.3390/ph19071096

**Published:** 2026-07-16

**Authors:** Chun-Sik Bae, Jin-Woo Park, Soon-Young Lee, So-Hyeon Bok, Seung-Yub Song, Dae-Hun Park, Seung-Sik Cho

**Affiliations:** 1 College of Veterinary Medicine, Chonnam National University, Gwangju 61186, Republic of Korea; csbae210@chonnam.ac.kr; 2 Bio-Medicine Advanced Formulation Research Center, RLRC, Mokpo National University, Muan 58554, Republic of Korea; jwpark@mnu.ac.kr (J.-W.P.); asy390@naver.com (S.-Y.L.); bok_23@naver.com (S.-H.B.); tgb1007@naver.com (S.-Y.S.); 3 Biomedicine, Health & Life Convergence Sciences, BK21 Four, College of Pharmacy, Mokpo National University, Muan-gun 58554, Republic of Korea; 4 College of Oriental Medicine, Dongshin University, Naju-si 58245, Republic of Korea

**Keywords:** manumycin A, DSS-induced colitis, inflammation, in vivo, mouse model

## Abstract

**Background/Objectives**: Manumycin A, a natural polyketide antibiotic isolated from Streptomyces species, has been reported to exhibit anticancer, anti-inflammatory, and immunomodulatory activities through regulation of multiple signaling pathways. However, its therapeutic potential in inflammatory bowel disease (IBD) has not yet been investigated. This study aimed to evaluate the protective effects of Manumycin A in a dextran sulfate sodium (DSS)-induced colitis mouse model. **Methods**: Experimental colitis was induced in male ICR mice by administration of 3% DSS in drinking water for 7 days. Manumycin A (1, 5, and 10 mg/kg) was administered via intraperitoneal injection, and 5-aminosalicylic acid (100 mg/kg) was used as a positive control. Disease severity was evaluated by body weight changes, disease activity index (DAI), colon length, histopathological analysis, and immunohistochemical assessment of pro-inflammatory cytokines, including IL-1β, IL-6, TNF-α, and IFN-γ. **Results**: Manumycin A treatment attenuated DSS-induced colitis in a dose-dependent manner. Although body weight changes were modest and did not show statistically significant differences among groups, Manumycin A significantly reduced DAI scores compared with the DSS-treated group. Treatment also alleviated DSS-induced colon shortening and improved histopathological alterations, including epithelial damage, mucosal disruption, and inflammatory cell infiltration. Immunohistochemical analysis showed that Manumycin A reduced the expression of IL-1β, IL-6, TNF-α, and IFN-γ in colon tissues. **Conclusions**: Manumycin A exerted protective effects against DSS-induced colitis by attenuating inflammatory responses and improving colonic tissue damage. These findings suggest that Manumycin A may have therapeutic potential as a candidate for the treatment of IBD.

## 1. Introduction

Manumycin A is a natural polyketide antibiotic belonging to the manumycin family, originally isolated from *Streptomyces* species, and is widely recognized as a potent inhibitor of farnesyltransferase [1]. It has been extensively studied for its anticancer properties, primarily through modulation of Ras signaling pathways, induction of apoptosis, and regulation of oxidative stress and inflammatory responses [2]. In addition to its effects on tumor cell proliferation, accumulating evidence suggests that manumycin A can influence key signaling pathways such as NF-κB and MAPK, which play critical roles in both tumor progression and inflammatory processes [3,4].

Ras proteins require post-translational farnesylation for proper membrane localization and activation of downstream signaling pathways. Farnesyltransferase-mediated Ras signaling has been implicated not only in tumorigenesis but also in the regulation of inflammatory responses through modulation of nuclear factor-kappa B (NF-κB), mitogen-activated protein kinase (MAPK), and cytokine-associated signaling cascades. Excessive activation of these pathways contributes to the production of pro-inflammatory mediators, including tumor necrosis factor-α (TNF-α), interleukin (IL)-1β, and IL-6, which are critically involved in the pathogenesis of IBD. Previous studies have demonstrated that manumycin A suppresses IκB kinase β activity and NF-κB signaling, suggesting that its biological activity may extend beyond tumor suppression to the regulation of inflammatory processes.

Despite these promising biological activities, most studies on manumycin A have been limited to in vitro systems or tumor xenograft models, with a primary focus on cytotoxicity and antitumor efficacy [5,6]. In vivo studies have generally demonstrated that manumycin A is tolerated within a dose range of approximately 1–5 mg/kg without severe toxicity [5]. However, its biological effects in non-cancer disease models remain largely unexplored.

IBD, including ulcerative colitis and Crohn’s disease, is characterized by chronic intestinal inflammation, epithelial barrier disruption, and excessive production of pro-inflammatory mediators. At the molecular level, intestinal inflammation is associated with dysregulated cytokine networks, activation of NF-κB and MAPK pathways, recruitment of immune cells, and disruption of epithelial barrier integrity. These events collectively contribute to tissue injury and disease progression, making them important therapeutic targets for the development of anti-inflammatory agents. Dextran sulfate sodium (DSS)-induced colitis is a well-established experimental model that reproduces key clinical and histopathological features of human IBD, including body weight loss, colon shortening, epithelial damage, and inflammatory cell infiltration [7].

Although various compounds have been investigated using DSS-induced colitis models, the effects of Manumycin A on intestinal inflammation have not yet been reported. Given its reported regulatory effects on inflammation-associated signaling pathways, including NF-κB and MAPK, we hypothesized that Manumycin A may exert protective effects against intestinal inflammation.

Therefore, the present study investigated the protective effects of Manumycin A in a DSS-induced colitis mouse model. To evaluate its in vivo pharmacological efficacy under controlled systemic exposure, Manumycin A was administered via intraperitoneal injection at doses of 1, 5, and 10 mg/kg. The effects of treatment were evaluated based on disease activity index, colon length, histopathological alterations, and inflammatory cytokine expression in colon tissues.

## 2. Results

### Effects of Manumycin A in a DSS-Induced Murine Colitis Model

**Figure 1:**
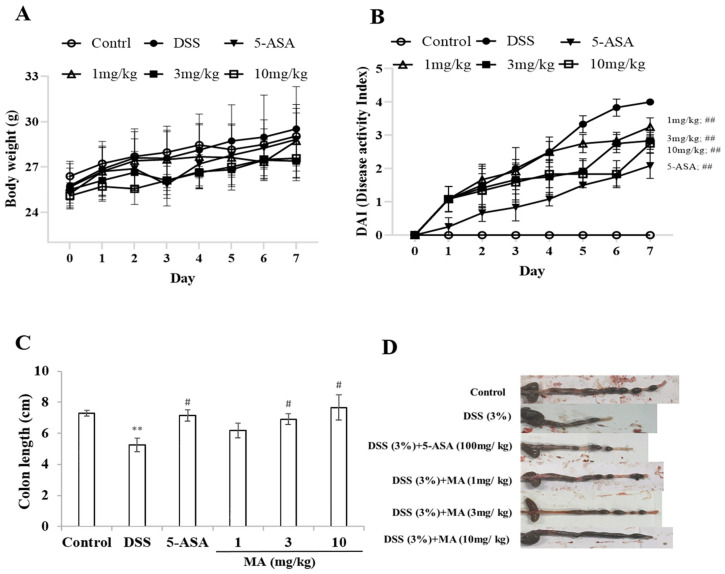
Preventive effects of Manumycin A (MA) on DSS-induced colitis in mice. (**A**) Body weight changes, (**B**) disease activity index (DAI), (**C**) colon length, and (**D**) representative images of colons from each group. MA treatment attenuated DSS-induced colitis, as evidenced by reduced DAI scores and improved colon length compared with DSS-treated controls. Body weight changes were monitored throughout the experimental period. Data are presented as mean ± SD (*n* = 6 mice per group). Statistical significance was defined as ** *p* < 0.01 versus the non-colitic group, and # *p* < 0.05 and ## *p* < 0.01 versus the DSS-treated group.

Manumycin A attenuated DSS-induced colitis in mice, as evidenced by improvements in disease activity index (DAI), colon shortening, histopathological alterations, and inflammatory cytokine expression. Body weight changes during the experimental period were relatively modest across all groups, and no statistically significant differences were observed (Figure 1A). However, Manumycin A-treated groups showed significantly reduced DAI scores compared with the DSS-treated group at Day 7 (Figure 1B), indicating attenuation of disease severity.

In addition, Manumycin A-treated mice exhibited significantly longer colons than DSS-treated controls (Figure 1C,D), indicating reduced colonic inflammation and disease severity.

**Figure 2:**
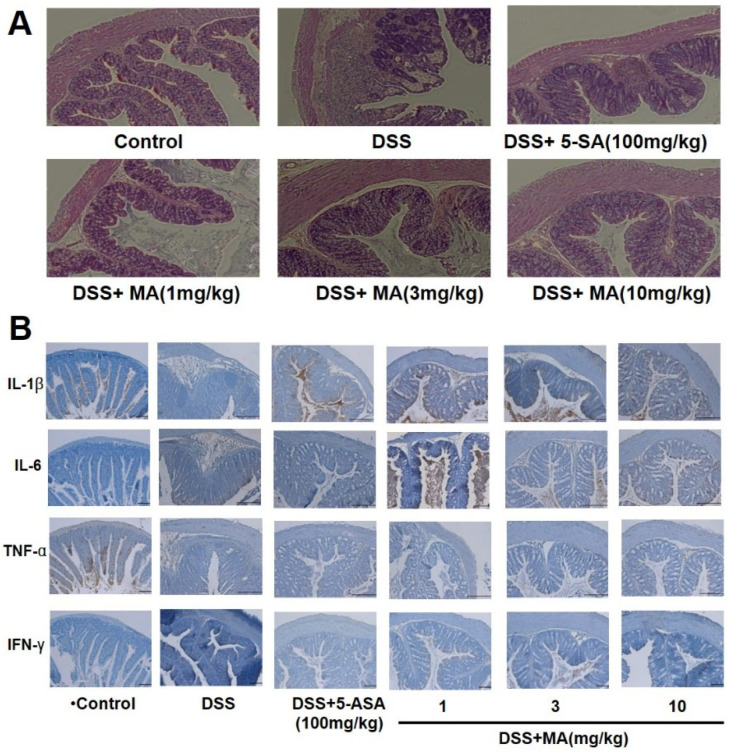
Effects of manumycin A (MA) on histological damage and inflammatory responses in DSS-induced colitis. (**A**) Representative hematoxylin and eosin-stained colon sections from control, DSS-induced colitis, 5-aminosalicylic acid (5-ASA, 100 mg/kg), and MA-treated groups (1, 3, and 10 mg/kg). DSS treatment induced marked epithelial damage, mucosal erosion, and inflammatory cell infiltration, whereas MA treatment markedly preserved tissue architecture and reduced inflammation. Magnification, ×100. (**B**) Immunohistochemical analysis of pro-inflammatory cytokines (IL-1β, IL-6, TNF-α, and IFN-γ) in colon tissues. MA treatment significantly reduced inflammatory responses, as evidenced by decreased cytokine expression.

Histological analysis further supported these findings. DSS-treated mice displayed severe epithelial damage, mucosal erosion, loss of glandular architecture, and extensive inflammatory cell infiltration. In contrast, treatment with Manumycin A dose dependently preserved colonic structure and reduced inflammatory infiltration (Figure 2A).

Furthermore, Manumycin A reduced the expression of pro-inflammatory cytokines, including IFN-γ, IL-1β, IL-6, and TNF-α, in colon tissues in a dose-dependent manner (Figure 2B), supporting its anti-inflammatory effects in DSS-induced colitis.

## 3. Discussion

In the present study, Manumycin A significantly attenuated DSS-induced colitis, as evidenced by improvements in disease activity index, colon shortening, histopathological damage, and inflammatory cytokine expression. Although previous studies have primarily focused on the anticancer activity of manumycin A in tumor models [5,6], the present findings demonstrate its protective effects in an experimental model of intestinal inflammation. These results expand the biological relevance of manumycin A beyond its previously reported anticancer applications.

A notable finding of the present study was the marked reduction in the expression of the pro-inflammatory cytokines IFN-γ, IL-1β, IL-6, and TNF-α. These cytokines are recognized as key mediators of intestinal inflammation and contribute to epithelial injury, immune cell recruitment, and disease progression in (IBD) [8]. Elevated levels of TNF-α and IL-6 have been consistently associated with disease severity in both experimental colitis and human IBD, while IL-1β and IFN-γ promote amplification of inflammatory responses and disruption of mucosal homeostasis. Therefore, suppression of these cytokines may represent an important mechanism underlying the protective effects of manumycin A observed in the present study.

The molecular basis of these effects may be associated with the well-characterized activity of manumycin A as a farnesyltransferase inhibitor [9]. Farnesylation is a critical post-translational modification required for the membrane localization and activation of Ras family proteins. Inhibition of Ras signaling has been shown to influence multiple downstream pathways involved in inflammation, including NF-κB and mitogen-activated protein kinase (MAPK) signaling [10]. Although Ras proteins have traditionally been studied in the context of oncogenic transformation, increasing evidence suggests that Ras-dependent signaling also participates in the regulation of innate and adaptive immune responses [11]. Consequently, pharmacological inhibition of farnesyltransferase may influence inflammatory processes independently of its anticancer activity.

Among the potential downstream mechanisms, NF-κB signaling represents a particularly attractive target. NF-κB functions as a master regulator of inflammatory gene expression and controls the transcription of numerous cytokines, chemokines, and adhesion molecules involved in intestinal inflammation. Previous studies have demonstrated that manumycin A can suppress IκB kinase β activity and inhibit NF-κB activation [3]. Such effects are consistent with the reduced expression of TNF-α, IL-1β, and IL-6 observed in the present study. Although NF-κB activation was not directly evaluated, the cytokine profile observed in colon tissues suggests that modulation of NF-κB-associated inflammatory signaling may contribute to the protective activity of manumycin A.

In addition to NF-κB, MAPK pathways may also be involved. The ERK, JNK, and p38 MAPK pathways are activated during DSS-induced intestinal injury and regulate the production of inflammatory mediators, immune cell activation, and tissue damage [12]. Previous investigations have reported that manumycin A can influence Ras-dependent MAPK signaling in several experimental systems. Given the established contribution of MAPK pathways to the pathogenesis of colitis, it is plausible that attenuation of MAPK-mediated inflammatory responses contributed to the reduced cytokine expression and histological damage observed in the present study. Future studies evaluating phosphorylation of ERK, JNK, and p38 would be valuable for clarifying this possibility.

Another important aspect of DSS-induced colitis is disruption of epithelial barrier integrity [13]. DSS directly damages the intestinal epithelium, resulting in increased permeability and exposure of the mucosal immune system to luminal antigens and microbial products. This process initiates a cascade of inflammatory events that ultimately leads to mucosal ulceration, inflammatory cell infiltration, and tissue destruction. The preservation of epithelial architecture and reduction of inflammatory infiltration observed following manumycin A treatment suggest that suppression of inflammatory mediators may indirectly contribute to maintenance of intestinal tissue integrity. Restoration of epithelial homeostasis represents an important therapeutic objective in IBD and may partially explain the beneficial effects observed in the present study.

In the present study, Manumycin A was administered via intraperitoneal injection to ensure consistent systemic exposure and minimize variability associated with oral absorption. Since the pharmacokinetic properties and oral bioavailability of Manumycin A remain largely unknown, this route was considered appropriate for evaluating its in vivo pharmacological efficacy in this initial proof-of-concept study. Although oral administration or colon-targeted delivery may provide greater clinical relevance for IBD, the present findings support the anti-inflammatory potential of Manumycin A under controlled experimental conditions.

An additional observation was the apparent tolerability of manumycin A at doses up to 10 mg/kg. Previous in vivo investigations have largely examined lower doses in tumor xenograft models, and information regarding its use in inflammatory disease models remains limited [9]. Although formal toxicity assessments were not performed in the present study, no obvious adverse effects were observed during the experimental period. These findings suggest that manumycin A may possess a favorable therapeutic profile within the tested dose range.

Several limitations should be acknowledged. First, the present study focused primarily on disease outcomes and inflammatory cytokine expression and did not directly evaluate molecular signaling pathways such as Ras, NF-κB, or MAPK. Therefore, the proposed mechanisms remain speculative and require experimental validation. Second, quantitative analyses of inflammatory mediators, such as serum cytokine measurement or ELISA-based assays, were not included in the present study. Although tissue-level cytokine expression was evaluated by immunohistochemistry, additional quantitative analyses would further strengthen the findings. Third, the findings were obtained using a single DSS-induced colitis model and may not fully represent the complexity of human IBD. Finally, long-term efficacy and safety were not investigated. Future studies incorporating molecular analyses, quantitative inflammatory marker assessments, and additional experimental models will be important for establishing the mechanistic basis and translational relevance of Manumycin A.

Collectively, the present findings demonstrate that Manumycin A exerts anti-inflammatory and tissue-protective effects in experimental colitis. The observed reduction in pro-inflammatory cytokine expression, together with the known regulatory effects of Manumycin A on Ras-associated signaling pathways, suggests that modulation of NF-κB and MAPK signaling may contribute to its biological activity. These findings broaden the pharmacological relevance of Manumycin A and support further investigation of its therapeutic potential as a candidate for IBD treatment.

## 4. Materials and Methods

### 4.1. Materials

Manumycin A was prepared from *Streptomyces* sp. CS392 using a previously described extraction and purification procedure (Figure S1) [14]. Briefly, culture broth was extracted with ethyl acetate and purified by silica gel chromatography followed by reverse-phase C18 column chromatography to obtain manumycin A.

### 4.2. DSS-Induced Colitis Model

All animal experiments were approved by the Institutional Animal Care and Use Committee (IACUC) of Chonnam National University (IACUC-YB-R-2019-49) and conducted in accordance with institutional guidelines for the care and use of laboratory animals. Male ICR mice (5 weeks old, approximately 25 g) were housed under standard laboratory conditions with free access to food and water.

Acute colitis was induced by administration of 3% dextran sulfate sodium (DSS; Sigma-Aldrich, St. Louis, MO, USA) in drinking water for 7 consecutive days. Mice were randomly assigned to five groups (*n* = 6 per group): a non-colitic control group, a DSS-treated control group, a 5-aminosalicylic acid (5-ASA, 100 mg/kg/day) group, and manumycin A-treated groups (1, 3, and 10 mg/kg/day). Both 5-ASA and manumycin A were administered intraperitoneally once daily for 7 days. As 5-ASA is widely used for the treatment of IBD, it was included as a positive control [15].

Disease activity index (DAI) was assessed daily based on body weight loss, stool consistency, and fecal bleeding according to previously described criteria [16].

At the end of the experimental period, mice were anesthetized with ketamine (100 mg/kg) and xylazine (10 mg/kg) administered intraperitoneally. Adequate anesthesia was confirmed by the absence of a pedal withdrawal reflex. The animals were then euthanized, and the colons were excised, rinsed with saline, and measured for length. Colon tissues were fixed in 10% formaldehyde, embedded in paraffin, sectioned, and stained with hematoxylin and eosin for histological evaluation.

### 4.3. Histopathological and Immunohistochemical Analysis

Immunohistochemical staining was performed as previously described [17]. Briefly, paraffin-embedded sections were deparaffinized and treated with 3% hydrogen peroxide in methanol for 10 min to block endogenous peroxidase activity. Antigen retrieval was performed in 0.1 M sodium citrate buffer. After blocking with normal serum, sections were incubated overnight at 4 °C with primary antibodies against IFN-γ (Santa Cruz, sc-74104, Santa Cruz, CA, USA), TNF-α (MY BioSource, MBS175453, San Diego, CA, USA), IL-1β (Santa Cruz, sc-1251), and IL-6 (Santa Cruz, sc-7920). The sections were then incubated with a biotinylated secondary antibody (1:500; DAKO, Glostrup, Denmark) followed by horseradish peroxidase-conjugated streptavidin. Immunoreactivity was visualized using 3,3-diaminobenzidine tetrahydrochloride and counterstained with Mayer’s hematoxylin. Images were captured using an Axioscope A1 microscope (Carl Zeiss, Oberkochen, Germany).

### 4.4. Statistical Analysis

Data are presented as the mean ± standard deviation (SD). Statistical analyses were performed using one-way analysis of variance (ANOVA) followed by Dunnett’s multiple comparison test. Statistical significance was defined as *p* < 0.05.

## 5. Conclusions

In conclusion, Manumycin A significantly attenuated DSS-induced colitis in mice, as demonstrated by improvements in disease activity index, colon length, histopathological alterations, and inflammatory cytokine expression. These findings suggest that Manumycin A exerts anti-inflammatory and tissue-protective effects in experimental colitis, potentially through modulation of inflammation-associated signaling pathways. Further studies are warranted to elucidate the underlying molecular mechanisms and to evaluate its therapeutic potential in IBD.

## Data Availability

The original contributions presented in this study are included in the article. Further inquiries can be directed to the corresponding author.

## Supplementary Materials

The following supporting information can be downloaded at https://www.mdpi.com/article/10.3390/ph19071096/s1, Figure S1: Structural characterization of Manumycin A. Representative spectroscopic data used for structural confirmation, including ^1^H NMR, COSY, HMQC, and TOCSY spectra. Figure S2: High-resolution representative H&E-stained colon tissue images. ×200, 100 μM (for Figure 2A). Figure S3: High-resolution representative immunohistochemical images showing IL-1β, IL-6, TNF-α, and IFN-γ expression in colon tissues. ×200, 100 μM (For Figure 2B). Figure S4: Experimental design and dosing regimen for Manumycin A treatment in the DSS-induced colitis model.
