# Supplementary material for: Manumycin A Attenuates DSS-Induced Colitis in Mice via Anti-Inflammatory Effects Following Intraperitoneal Administration
Source: Pharmaceuticals (Basel). 2026 Jul 16;19(7):1096. doi: 10.3390/ph19071096 (PMC13414652; doi:10.3390/ph19071096)

Figure S1. Structural characterization of Manumycin A. Representative spectroscopic data used for structural confirmation, including ^{1}H NMR, COSY, HMQC, and TOCSY spectra.

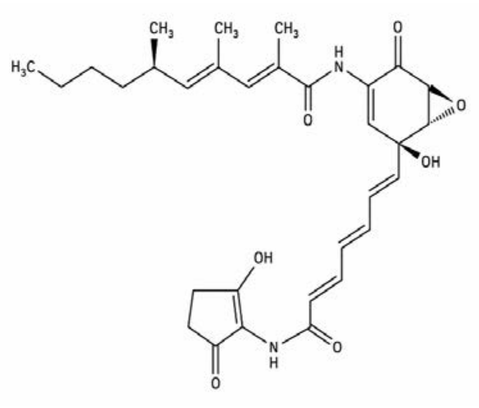

chemical structure of Manumycin A

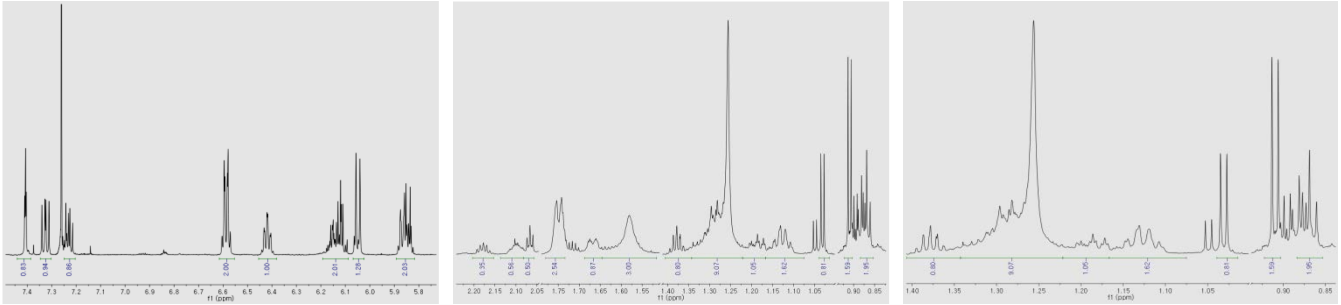

^{1}H NMR

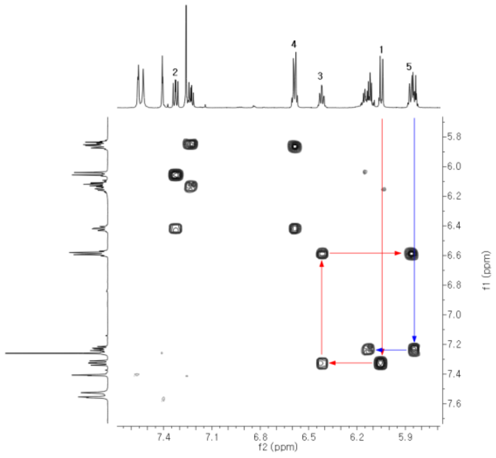

COSY

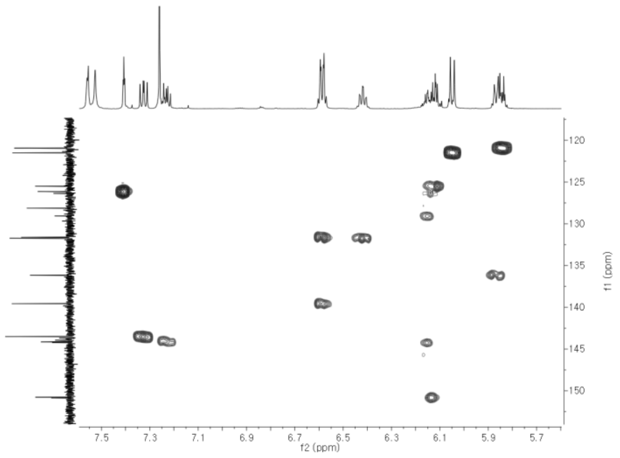

HMQC

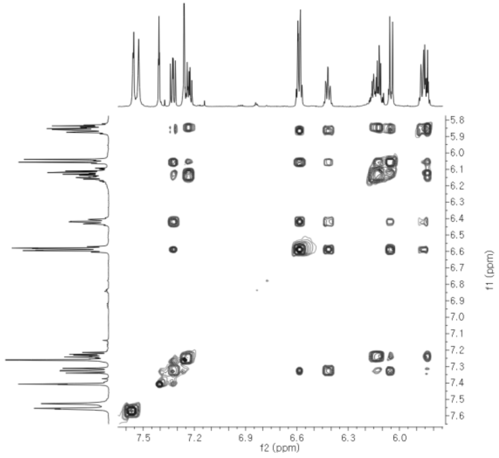

TOCSY

Figure S2. High-resolution representative H&E-stained colon tissue images.**x**200, 100 **μ**M (for figure 2A)

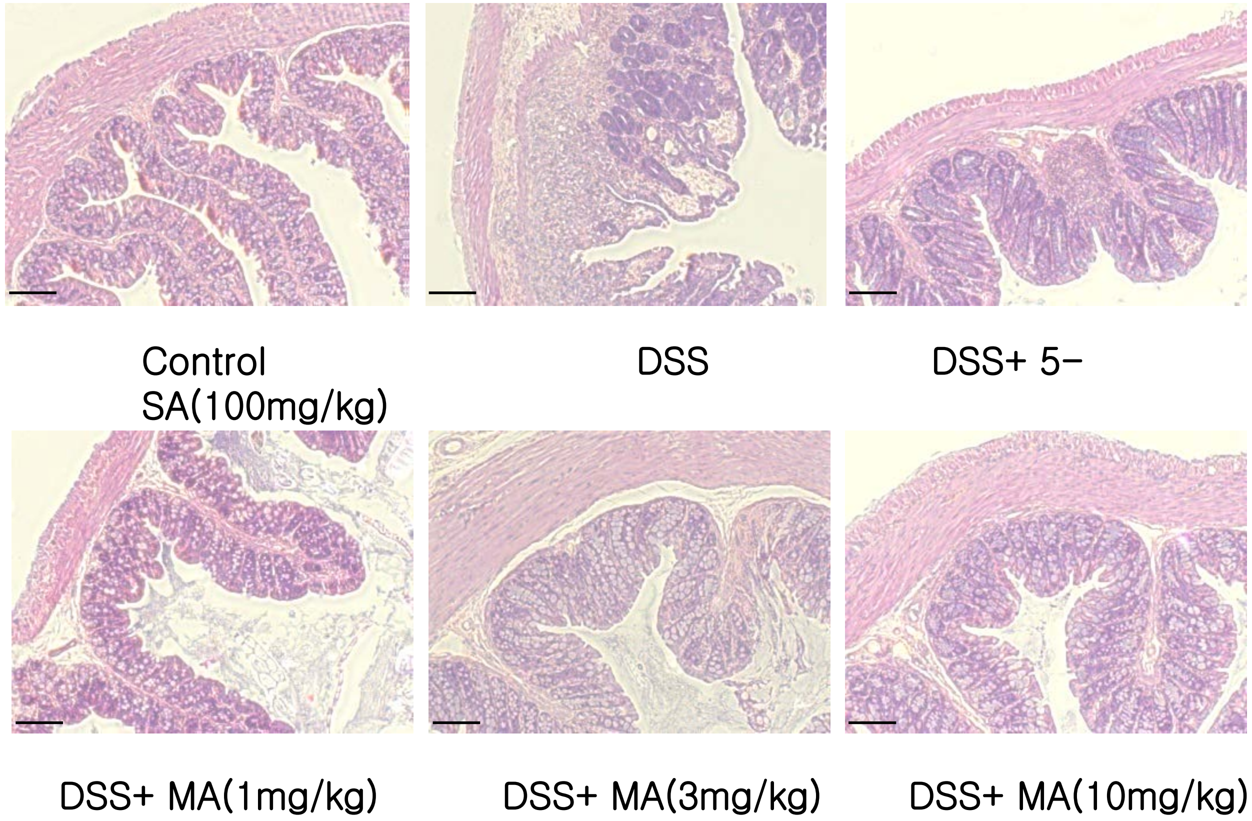

Figure S3. High-resolution representative immunohistochemical images showing IL-**1β, IL**-6, TNF-**α, and IFN**-**γ expression in colon tissues. x**200, 100 **μ**M (For figure 2B)

Goto next page

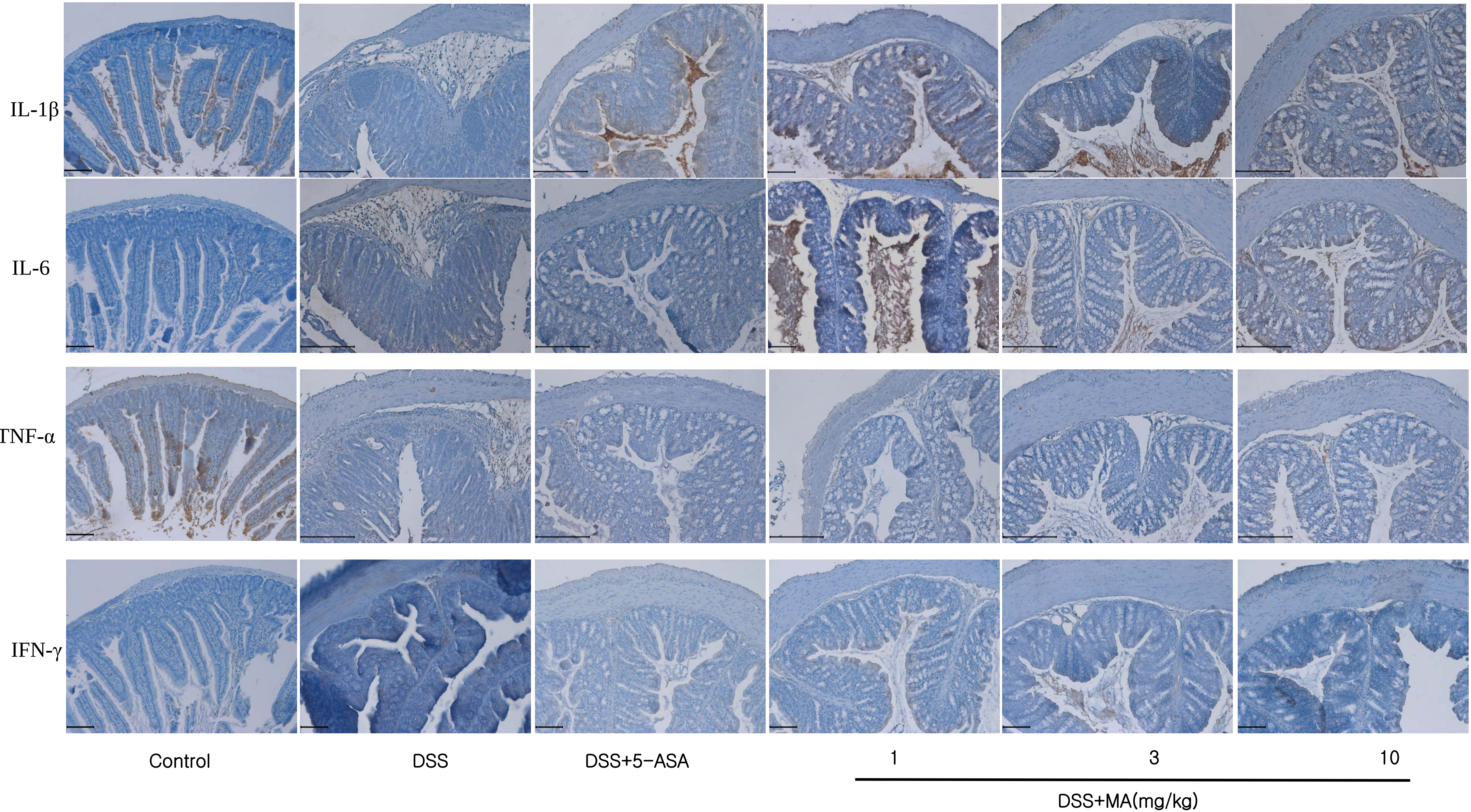
IL-1β
IL-6
TNF-α
IFN-γ
Control
DSS
DSS+5-ASA
1
3
10
DSS+MA(mg/kg)

Figure S4 Experimental design and dosing regimen for Manumycin A treatment in the DSS-induced colitis model.

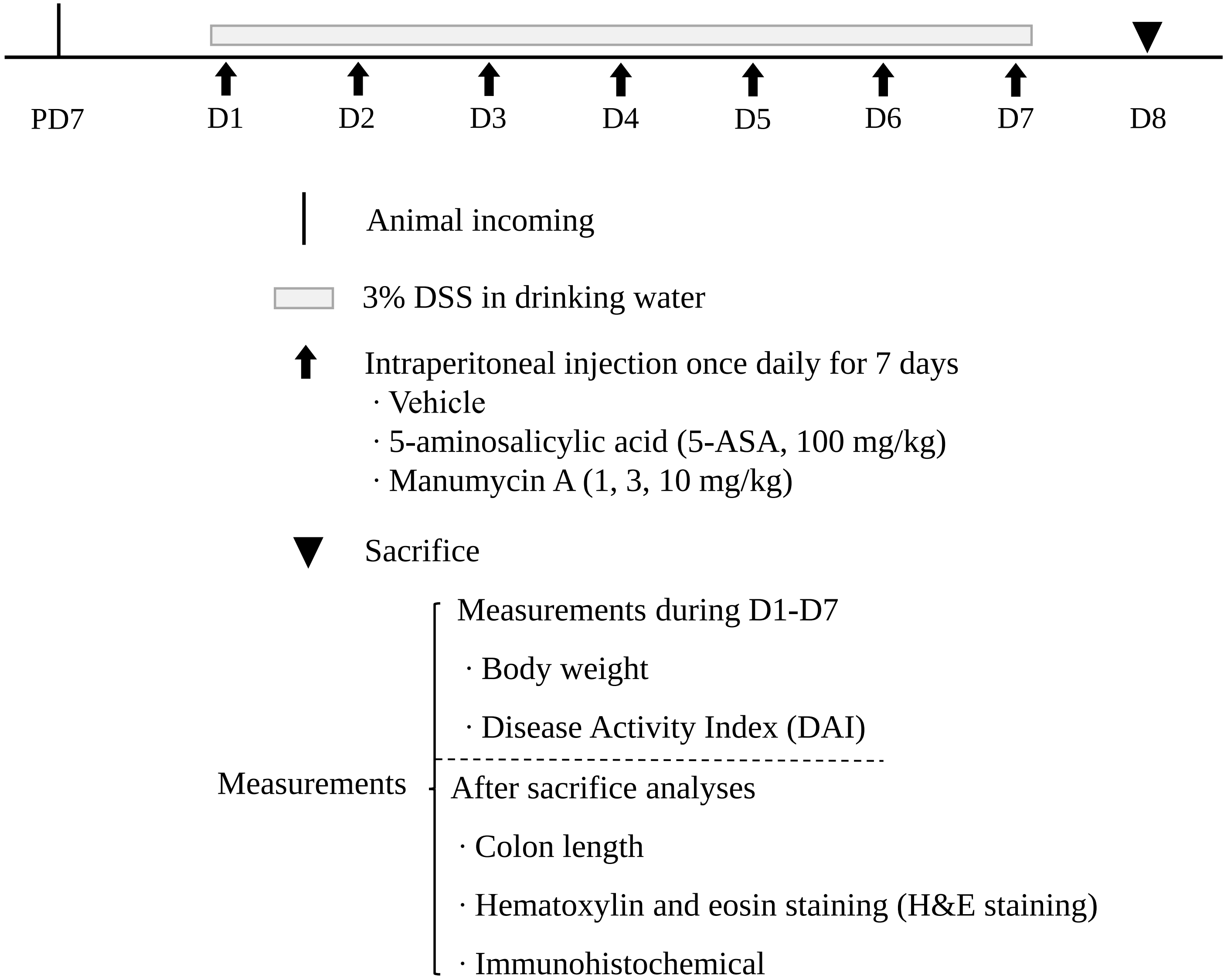

Supplement: Supplementary file 1 [file pharmaceuticals-19-01096-s001.zip › pharmaceuticals-4394418-supplementary.pdf]
